# Renal secondary hyperparathyroidism should be considered a differential diagnosis in forensic cases where animal abuse is suspected

**DOI:** 10.1007/s12024-022-00501-5

**Published:** 2022-08-19

**Authors:** Leonie W. L. van Bruggen, Javier P. Deniz Marrero, Guy C. M. Grinwis, Nienke Endenburg, Reza R. R. Gerretsen, Hubert G. T. Nijs

**Affiliations:** 1 Division Integrating Disciplines (Diagnostic Imaging), Department Clinical Sciences, Faculty of Veterinary Medicine, Utrecht University, Utrecht, The Netherlands; 2 Division Pathology, Department Biomolecular Health Sciences, Faculty of Veterinary Medicine, Utrecht University, Utrecht, The Netherlands; 3 Division Animals in Science and Society, Department of Population Health Sciences, Faculty of Veterinary Medicine, Utrecht University, Utrecht, The Netherlands; 4 Division of Special Services and Expertise, Netherlands Forensic Institute (NFI), The Hague, The Netherlands

**Keywords:** Animal abuse, Veterinary forensics, Diagnostic imaging, Renal secondary hyperparathyroidism

## Abstract

This case description reports an adult dog with multiple rib fractures that was initially suspected to be the victim of non-accidental trauma, but eventually was diagnosed with severe chronic interstitial nephritis and renal secondary hyperparathyroidism leading to fibrous osteodystrophy and multiple pathological fractures. The importance of the specific expertise of a dedicated expert panel on animal abuse is discussed. This case illustrates the significance of the identification of normal, pathological, and breed-related variations within an animal in addition to forensic expertise, emphasizing that the presence of multidisciplinary teams in an expert panel on animal abuse is crucial.

## Introduction

Veterinary forensic imaging is an emerging field of expertise [1, 2].

Distinguishing traumatic from non-traumatic injury is important and sometimes difficult in both human and veterinary medicine. In human medicine, expert knowledge of detection of child abuse is made available to healthcare workers through Expert Panel Consultation Teams, in the Netherlands organized via the Dutch Expert Centre for Child Abuse [3, 4]. Similar to the human counterpart, an expert panel for animal abuse was launched in 2017 to provide expert assistance to veterinary clinicians in cases where animal abuse is suspected: the Veterinary Forensic Expert Centre (VFEC) [5].

In this paper we report a case of suspected animal abuse, where the referring veterinarian consulted the VFEC, and the course of things thereafter.

## Case report: initial presentation

A 3.5-year-old female spayed Shih Tzu dog was presented to its local veterinarian with signs of respiratory problems when in recumbent position. The patient was up to date with vaccinations and had a dental treatment with some extractions 8 months prior to current presentation. No other health problems were mentioned in the patient’s history. The dog stayed 10 days at a boarding kennel 3 months prior to presentation and had been sneezing since. On presentation, the dog was alert but a bit shaky and showed no abnormalities while exploring the consulting room. The dog was malnourished, according to its owner she never ate well, and now also showed weight loss. Physical examination showed poor dental hygiene, with tartar and gingivitis. The dog also showed respiratory distress with a stridor and occasionally open mouth breathing. Previous therapy with an anti-inflammatory agent and doxycycline had little effect and the stridor continued and hindered a thorough lung auscultation. Radiographs were made by the referring veterinarian. No blood tests were performed at this time.

On the acquired radiographs (2 lateral and 1 dorsoventral views of the thorax), the referring veterinarian noticed 2 fractured ribs (right 6th and 7th ribs) to which the owner reacted very emotional, stating to be “relieved that only a rib fracture was found and not a cardiac disease.” The owner could not explain when or how the ribs were fractured and declined the suggestion to contact the boarding kennel for more information. The referring veterinarian requested a colleague to interview the owner. This second veterinarian also felt uncomfortable with the response of the owner and advised her colleague to consult the VFEC.

## VFEC consultation

After receipt of the digital consultation form, including 3 radiographs in JPEG format, a panel of 3 expert members of the VFEC (an ECVDI^1^ diplomate and 2 forensic specialist physicians) was appointed by the coordinator of the VFEC and reviewed the case independently.

Assessment of the supplied radiographs revealed a coarse, artifactual haze in both the skeletal and soft tissue structures, complicating careful assessment of the fractures. The skeletal structures had a “mottled” appearance, and it was uncertain whether this was due to suboptimal technique or represented true pathology. Also, a diffuse, unstructured, interstitial pattern was noted throughout the lung field, further compromising accurate assessment of the fracture margins. Despite complicating technical artifacts, multiple fractures were noted by the panel in addition to the 2 fractures mentioned by the referring veterinarian, in at least 5 ribs (right 6th, 7th, 12th, and 13th ribs and one of the 10th ribs; fracture only visible on the lateral view and due to obliquity of the radiograph uncertain which side was involved) and suspected a fracture in one of the 9th ribs. Also a mild pectus carinatum was noted.

The fracture in the 13th rib (with broadening of the fracture ends) indicated a different stage of healing compared to the other fractured ribs. This, together with the location of the rib fractures, initially created support in the expert panel for the suspicion by the referring veterinarian of animal abuse. However, because the image quality of the 3 radiographs was considered suboptimal due to compression artifacts related to the JPEG format, and to better establish the role of technique and evaluate the presence of underlying pathology, original images in “Digital Imaging and Communications in Medicine” (DICOM) format were requested for review by the panel.

**Fig. 1:**
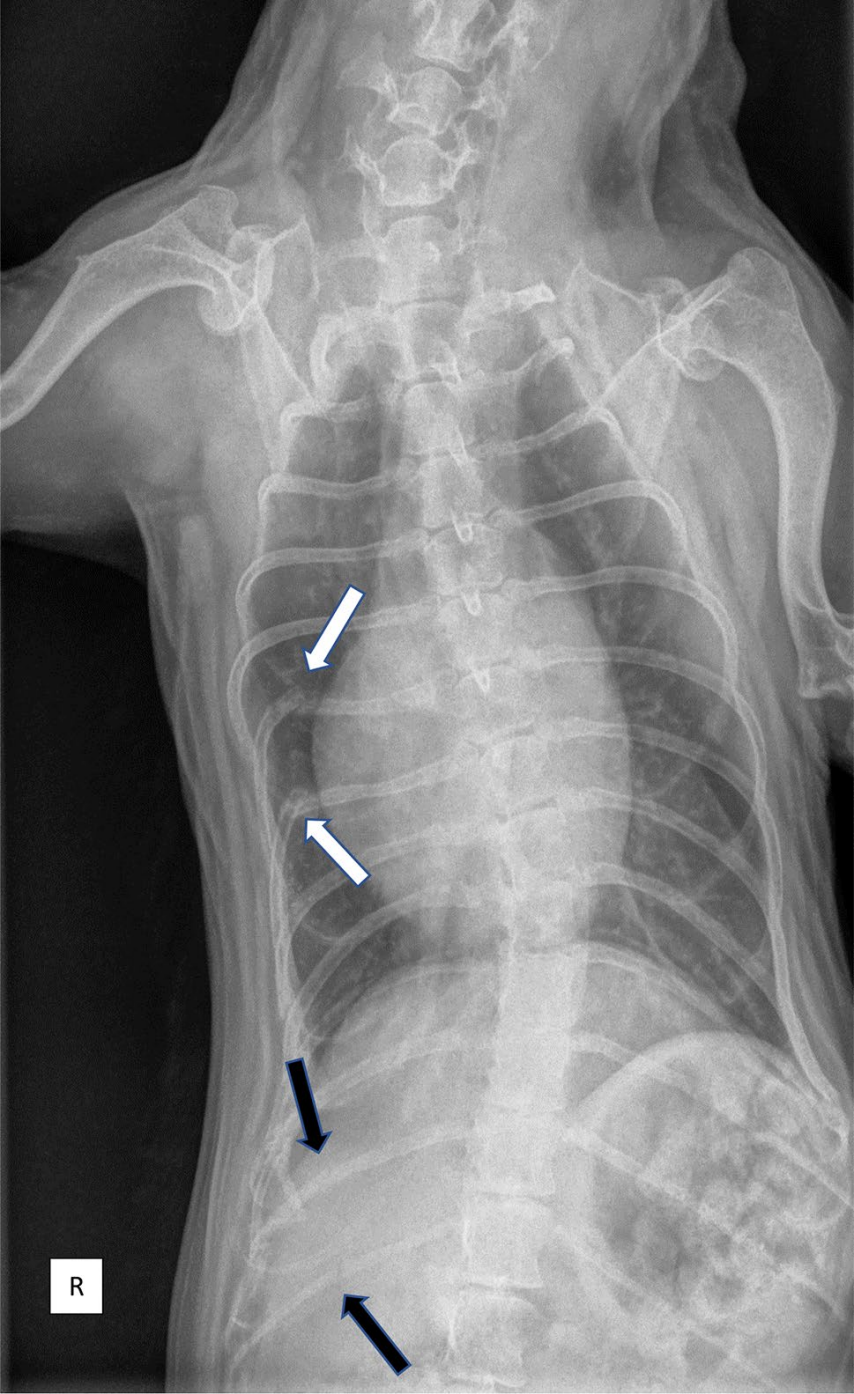
VD view of the thorax (R indicates right side of the dog). White arrows indicate the 2 fractures noted by the referring veterinarian. Black arrows indicate 2 additional fractures noted by the panel on this view

**Fig. 2:**
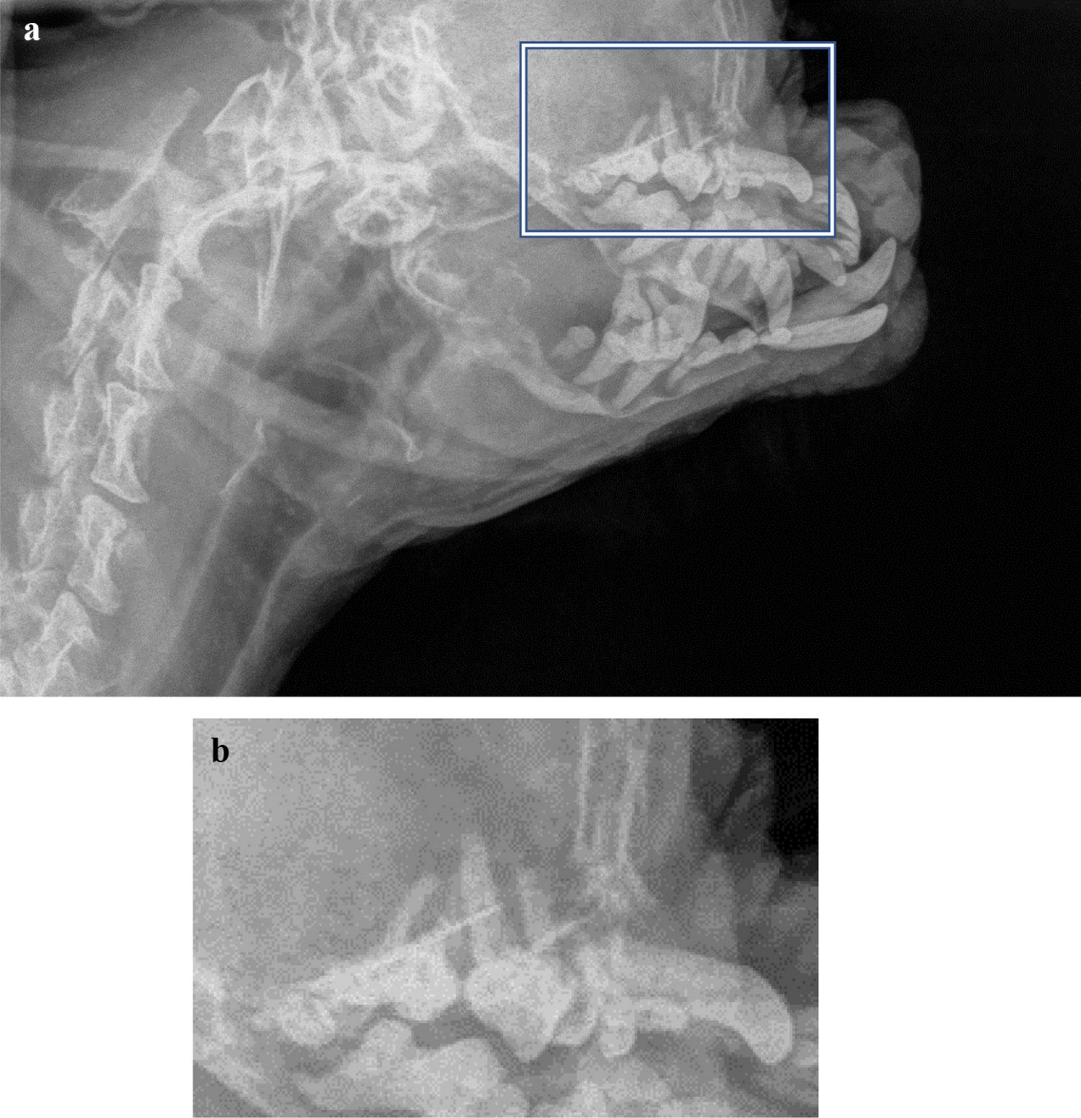
**a** Lateral view of the neck and part of the skull. **b** Detail of the maxilla. Notice the “floating teeth” appearance of multiple elements, secondary to the resorption of the lamina dura surrounding the tooth roots

In addition to the initially three submitted radiographs (Fig. 1), a fourth radiograph was added to the study, which included part of the skull (Fig. 2). This fourth radiograph revealed severe osteopenia of the skull, with loss of the lamina dura resulting in the suggestion of “floating teeth” which is considered to be highly suggestive of renal secondary hyperparathyroidism in dogs [6]. Additional to the findings in the skull, the DICOM images also confirmed a very mottled appearance of the skeletal structures, consistent with an osteopenic status of the complete skeleton.

The panel concluded the rib fractures to be most likely pathological of origin, secondary to renal secondary hyperparathyroidism, and this outcome was communicated to the referring veterinarian.

A blood test, performed subsequently by the referring veterinarian, confirmed the presence of a severe renal disease. Seventeen days after the initial consultation by the referring veterinarian, the dog deteriorated and the owner elected euthanasia. The owner consented to postmortem computed tomography (PMCT) and autopsy.

**Fig. 3:**
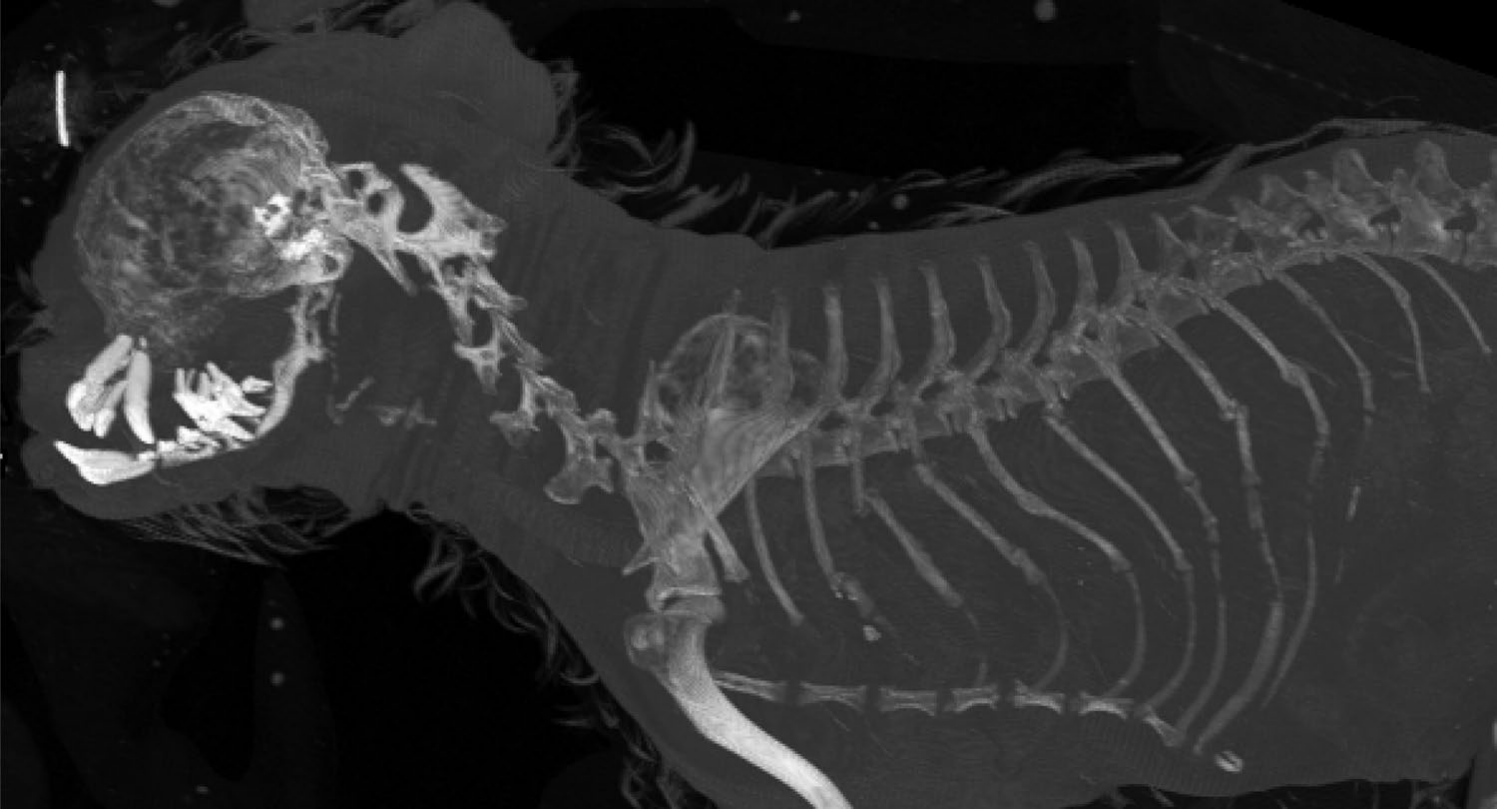
CT reconstruction of skull, neck, and thorax (right sided ribs and right foreleg only). Image is optimized for display of skeletal structures (bone window). Image shows multiple fractured ribs and the lack of calcium in scapula and skull, causing the suggestion of “floating teeth”

PMCT showed rib fractures in an additional 10 ribs, some ribs with multiple fractures, severe osteopenia of especially the mandible (consistent with the “rubber jaw”), and a generalized, mottled appearance of the skeletal structures due to resorption of calcium (Fig. 3). Findings also included increased soft tissue opacity in the nasal cavity and nasopharynx, small and irregularly formed kidneys, and small calcifications throughout the lungs.

**Fig. 4:**
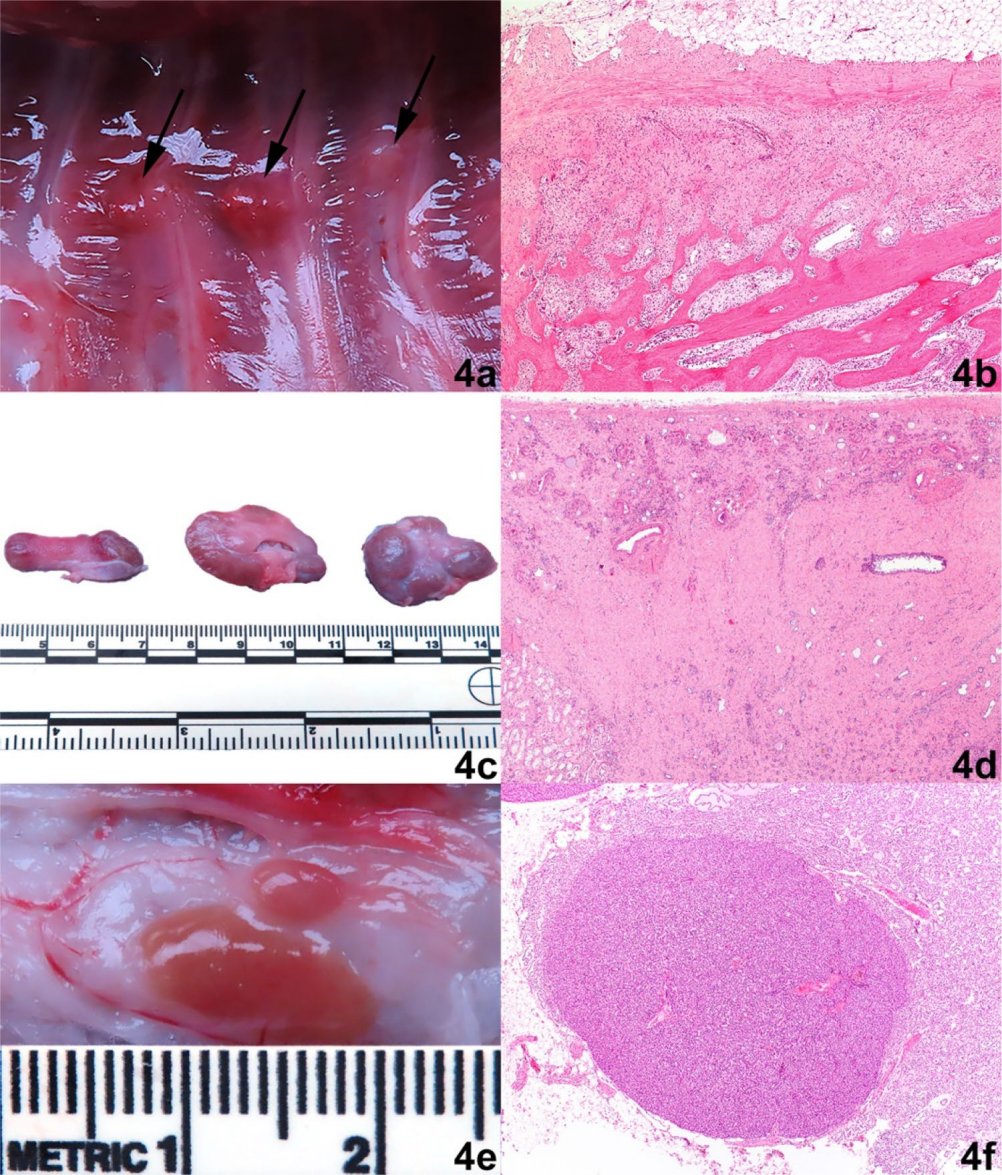
Macroscopical and histological pictures (hematoxylin and eosin stain) of lesions found in the Shih Tzu dog. **a** Multiple rib fractures of varying age (arrows). **b** Histological image of a fractured rib showing changes associated with osteopenia, fibrous osteodystrophy, and reactive bone proliferation. **c** Gross images of both kidneys. Left is the lateral view of the left kidney. In the center the cut surface of the right kidney and to the right the lateral view of the right kidney. Note the general small-size and highly irregular surface of the kidney. The indented, paler areas represent regions with extensive fibrosis and loss of functional renal tissue. **d** Histological image of the kidney. Severely thinned cortex is situated in the top section of the picture and is characterized by chronic inflammatory changes and extensive fibrosis which is also present in the medullary region of the kidney (bottom 2/3 of the image). **e** Enlarged parathyroid above the thyroid gland. **f** Enlarged parathyroid gland due to hypertrophy and hyperplasia of chief cells

Autopsy was performed the day after euthanasia. Multiple healed rib fractures consistent with findings at diagnostic imaging were noted (Fig. 4a), as well as flexible and fragile skull bones, scapula, and ribs that were also characterized by thickness reduction of bone. All maxillary teeth were mobile in the alveoli. Histologically, cortical and trabecular bones showed signs of osteopenia, fibrous osteodystrophy, and reactive bone proliferation (Fig. 4b). Both kidneys were macroscopically marked, diffuse reduced in size with multifocal areas of fibrosis (Fig. 4c). Histologically, the kidneys showed marked interstitial inflammation, glomerulosclerosis, and fibrosis of the renal tissue (Fig. 4d). The parathyroids were bilaterally enlarged (Fig. 4e, f), and the lungs were poorly collapsed and when compressed, did not recover their normal architecture (consistent with a pulmonary mineralization which was confirmed histologically). Additionally, the pleura over the cranial intercostal muscles showed multifocal areas compatible with mineralization.

## Discussion

Chronic renal failure is a fairly common problem in dogs, and renal secondary hyperparathyroidism is a well-known complication of this disease complex. Severe chronic renal disease in dogs can induce a progressive calcium-phosphorus imbalance leading to metastatic calcification of tissues. In order to maintain normal calcium concentrations in the blood, activation of parathyroid glands, as grossly depicted by an increase in size, results in elevated levels of parathyroid hormone with a subsequent increase in osteoclastic activity, leading to severe osteopenia with friable bones, facilitating the occurrence of pathological fractures [7–9]. The post-mortem findings in this dog confirmed the presence of a chronic interstitial nephritis with subsequent secondary hyperparathyroidism leading to fibrous osteodystrophy and bone fractures. The respiratory signs that were reported by the owner might be related to discomfort caused by the fractured ribs and the deposition of calcium within the alveolar walls. Renal secondary metabolic bone disease should be considered a differential diagnosis in suspected abuse cases in veterinary patients [6, 10–13].

Recognizing animal abuse is of importance in the context of not only animal welfare but also because of the relationship between animal abuse and domestic violence [14–19]. Consultation of an expert panel has shown added value in human medicine when dealing with suspected child abuse cases [4, 20, 21]. In this case, the added value of consulting the veterinary forensic expert panel was shown by the recognition of multiple, additional fractures, determining the negative effect of the use of compressed images of radiographs on image interpretation and identifying the pathological origin of the fractures. Furthermore, knowledge of a characteristic veterinary disease proved to be an essential asset in the interpretation of this case. Though renal secondary hyperparathyroidism in this dog does not rule out potential abuse as a cause for rib fractures, the panel considered it to be the most likely cause of the fractures in this dog given the presence of the disease and the severity of the changes noted in combination with the lack of other indications for abuse.

DICOM is the preferred image format when evaluating digital radiographs [22, 23]. In veterinary practice JPEG is sometimes used as alternative as they allow rapid transmission of large files to an off-site radiologist [24]. In first-line veterinary practice, due to cost restraints consumer-grade color LCDs, that are suboptimal for detailed image evaluation, are often used for soft-copy interpretation of radiographs [25]. For the subtle changes suspected on the initially supplied JPEG images of this dog, the image quality was deemed suboptimal and insufficient to answer the questions of the referring veterinarian by the radiologist; therefore, the original DICOM images were requested. These images were reviewed on a diagnostic monitor by the radiologist, confirming that the irregularities in skeleton and lungs represented true pathology and were not technique related. Additionally, the radiograph of the skull, initially not provided for consultation, confirmed the suspicion of an underlying pathology to the radiologist. This, together with the other images, eventually in DICOM, significantly altered the final advise of the panel. In general, great care should be taken to avoid errors based on the interpretation of images of suboptimal quality and DICOM currently is the gold standard in diagnostic image interpretation.

As reported in human (forensic) imaging, also in this patient CT proved to have additional value to radiography. CT revealed additional fractures and skeletal irregularities, renal changes, and pulmonary calcifications. However, 25 days had elapsed between the moment the radiographs were taken and the moment the PMCT was performed, hence it is possible that some fractures originate from this period.

In line with the expert panel on child abuse for humans, the veterinary counterpart Veterinary Forensic Expert Centre was launched. Both board-certified veterinary specialists and human experts in the field of forensic medicine and anthropology participate in the expert panel of the VFEC. In this case report, knowledge about key characteristics of renal secondary hyperparathyroidism in dogs, and technical limitations regarding image format and quality of the monitor used in image interpretation were essential in rendering a correct advice. Recognition of normal, pathological, and breed-related variations within an animal species, in addition to forensic expertise, requires the involvement of representatives of both domains in the VFEC and was found to be crucial by the authors in multiple cases where the VFEC was consulted so far.

## Key points

1. An accurate differential diagnosis in veterinary cases where animal abuse is suspected should be made by veterinary specialists
2. Consulting a dedicated expert panel on animal abuse cases showed to be of additional value, leading to a correct and timely diagnosis and avoiding an unfounded claim of animal abuse
3. Though veterinary forensics should be performed by veterinarians with relevant expertise, cooperation with human forensic experts was found to be beneficial, by both parties, and will help improve veterinary forensics as a whole

## Data Availability

Data available on request.

## Footnotes

1 European College of Veterinary Diagnostic Imaging. www.ECVDI.org
